# Design and Update of a Classification System: The UCSD Map of Science

**DOI:** 10.1371/journal.pone.0039464

**Published:** 2012-07-12

**Authors:** Katy Börner, Richard Klavans, Michael Patek, Angela M. Zoss, Joseph R. Biberstine, Robert P. Light, Vincent Larivière, Kevin W. Boyack

**Affiliations:** 1 Cyberinfrastructure for Network Science Center, School of Library and Information Science, Indiana University, Bloomington, Indiana, United States of America; 2 Royal Netherlands Academy of Arts and Sciences (KNAW), Amsterdam, The Netherlands; 3 SciTech Strategies, Inc., Berwyn, Pennsylvania, United States of America; 4 École de Bibliothéconomie et des Sciences de L’information, Université de Montréal, Montréal, Canada; 5 Observatoire des Sciences et des Technologies (OST), Centre Interuniversitaire de Recherche sur la Science et la Technologie (CIRST), Université du Québec à Montréal, Montréal, Canada; 6 SciTech Strategies, Inc., Albuquerque, New Mexico, United States of America; University of Illinois-Chicago, United States of America

## Abstract

Global maps of science can be used as a reference system to chart career trajectories, the location of emerging research frontiers, or the expertise profiles of institutes or nations. This paper details data preparation, analysis, and layout performed when designing and subsequently updating the UCSD map of science and classification system. The original classification and map use 7.2 million papers and their references from Elsevier’s Scopus (about 15,000 source titles, 2001–2005) and Thomson Reuters’ Web of Science (WoS) Science, Social Science, Arts & Humanities Citation Indexes (about 9,000 source titles, 2001–2004)–about 16,000 unique source titles. The updated map and classification adds six years (2005–2010) of WoS data and three years (2006–2008) from Scopus to the existing category structure–increasing the number of source titles to about 25,000. To our knowledge, this is the first time that a widely used map of science was updated. A comparison of the original 5-year and the new 10-year maps and classification system show (i) an increase in the total number of journals that can be mapped by 9,409 journals (social sciences had a 80% increase, humanities a 119% increase, medical (32%) and natural science (74%)), (ii) a simplification of the map by assigning all but five highly interdisciplinary journals to exactly one discipline, (iii) a more even distribution of journals over the 554 subdisciplines and 13 disciplines when calculating the coefficient of variation, and (iv) a better reflection of journal clusters when compared with paper-level citation data. When evaluating the map with a listing of desirable features for maps of science, the updated map is shown to have higher mapping accuracy, easier understandability as fewer journals are multiply classified, and higher usability for the generation of data overlays, among others.

## Introduction

Cartographic maps of physical places have guided humankind’s explorations for centuries. In addition to supporting navigation, these maps are used to record national boundaries or mineral resources, to show flows of trade activity, or to communicate areas of political unrest. Science maps of abstract semantic spaces aim to serve today’s explorers navigating the world of knowledge. These maps are generated through a scientific analysis of large-scale scholarly datasets in an effort to extract, connect, and make sense of the bits and pieces of knowledge they contain [1], [2]. Science maps can be used to gain overviews of “all-of-science” or of a specific subdiscipline. Science maps in combination with a mapping process for new datasets can be used to visually depict and compare data overlays, e.g., of funding vs. publication data [3]. Science maps can help identify major research areas, experts, institutions, collections, grants, papers, journals, and ideas in a domain of interest. They can show homogeneity vs. heterogeneity, cause and effect, and relative speed of progress. They allow us to track the emergence, evolution, and disappearance of topics and help to identify the most promising areas of research.

### 1.1 Related Work

Reviews of science mapping efforts up until 2007 show more than 200 different maps [4]. The number, diversity, and sophistication of science mapping efforts has increased enormously since then due to the availability of scholarly data in digital format, algorithm development, and an increase in computing power, see Mapping Science exhibit maps (http://scimaps.org). Each science map depicts an abstract high-dimensional space using different datasets, reference systems, and graphic designs. Very few maps depict all major disciplines of scholarly activity–these are also called global maps of science [5]. Some of these maps are drawn by hand while others are computer generated. Some sketch the expertise of one individual while others plot millions of data records from diverse sources. Many different data analysis and layout algorithms are used to arrive at two-dimensional representations of science. For example, the 1996 Map of Science by Small [6] used a combination of fractional citation counting and co-citation clustering via multidimensional scaling to extract a four level map from the 1996 Science Citation Index. Boyack and Klavans and colleagues created four global science maps: The Backbone of Science, the 2002 Base Map, the Paradigm Map, the UCSD Map of Science, see discussion and comparison in [4]. Leydesdorff and Rafols [5] used Journal Citation Report data covering journals included in the Science Citation Index (SCI) to create a map of Thomson Reuters subject categories and associated journals. Moya-Anegón et al. at SCImago aimed to create a graphic representation of the Spanish Science Research [7].

**Figure 1:**
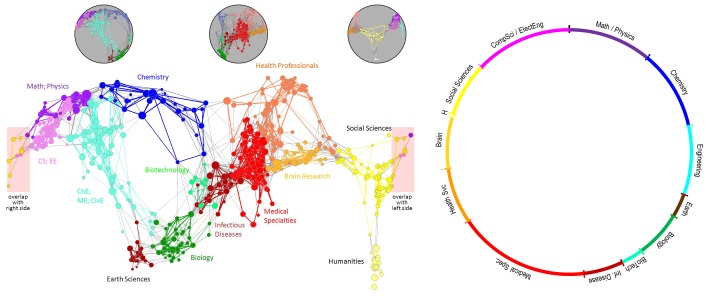
Visualizations of the UCSD Map: 2D Mercator projection (left) with three 3D spherical insets (top), 1D circular map (right). Note that the left hand side of the Mercator map connects to the right hand side.

Recent work by Klavans and Boyack compares the structure of 20 global maps of science [8]. They arrive at a consensus map generated from edges that occur in at least half of the input maps. The consensus map has a circular form connecting major disciplines of science in the following ordering: “mathematics is (arbitrarily) placed at the top of the circle, and is followed clockwise by physics, physical chemistry, engineering, chemistry, earth sciences, biology, biochemistry, infectious diseases, medicine, health services, brain research, psychology, humanities, social sciences, and computer science. The link between computer science and mathematics completes the circle.” Users need to learn how to read these maps–just like they learned the map of the world in school–before they can exploit these semantic reference systems to make sense of data overlays.

This paper opens by motivating the creation and usage as well as desirable properties of a general classification system and base map of science. Section 3 introduces methods applied to create the original UCSD map of science and methods applied to update it together with achieved results, complete with use cases. Section 4 presents a comparison of the original and the updated map and an evaluation of map properties. Section 5 concludes the paper with a discussion and outlook.

**Figure 2:**
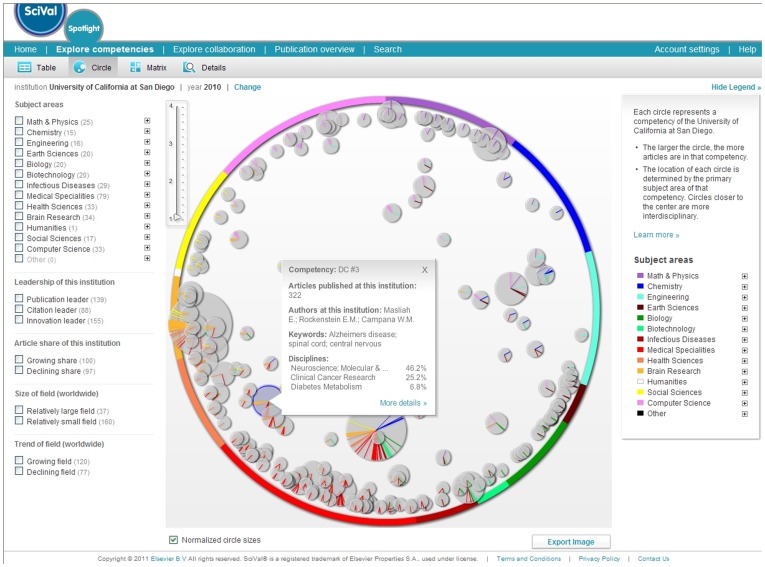
SciVal Spotlight map of one institution, here UCSD, showing institutional competencies. Each node within the circle map represents a competency (a group of linked topics), and is positioned at the average location of its articles. Node size reflects the number of articles. Coloured rays within each node show the disciplines that contribute to the competency.

**Figure 3:**
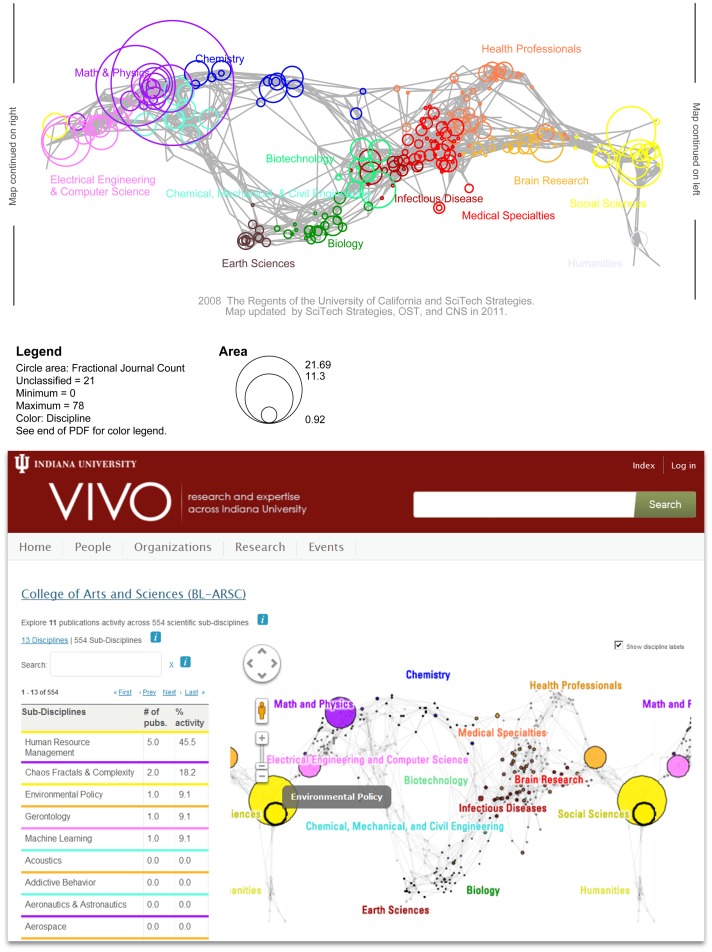
UCSD Map of Science visualizations in Sci2 desktop tool (top) and on VIVO web page (bottom). The left map shows the expertise coverage of four network science researchers, see Sci2 tutorial for details. The right map features the expertise coverage of the College of Arts and Sciences at Indiana University Bloomington. The interactive map is available online at http://vivo.iu.edu/vis/map-of-science/BL-ARSC).

### 1.2 User Needs and Research Questions

When used as a tool to grasp crucial developments in science and technology, a map of all of science holds potential value for a wide range of professionals. Among others, it can support knowledge and expertise exploration, navigation, management, and communication. Exemplary user groups and information seeking tasks comprise:

- **Students.** Maps of science can help students gain an overview of a particular knowledge domain, identify major research areas, experts, institutions, grants, publications, patents, citations, and journals as well as their interconnections, see the influence of certain theories, and gain a global picture of the domain.
- **Researchers.** Science maps can be used to ease access to research results, relevant funding opportunities, and potential collaborators [9] inside and outside the fields of inquiry, and to detect social networks and invisible colleges [10].
- **Grant Agencies/R&D Managers.** While maps of science cannot substitute for informed peer evaluation or expert panels, they can be used as tools to monitor (long-term) money flow and research developments, evaluate funding strategies for different programs, make informed decisions on project durations, and study funding patterns. In addition, they can also be used to identify the impact of research funding programs [11]–[14], scientific frontiers [15]–[18], the dynamics (speed of growth, diversification) of scientific fields [19], and complementary capabilities.
- **Industry/National Security Agency.** Maps of science can be utilized to gain access to major scientific results, knowledge carriers, etc. Information on needed technologies could be incorporated into maps, facilitating industry pulls for specific directions of research.
- **Data Providers.** Maps provide unique visual interfaces to digital libraries [20]. A successful example is the visual classification taxonomy developed and used in the MACE project. The portal [21] shows a birds-eye view of the hierarchical structure of over 2800 terms for tagging resources, allowing users to search and browse thousands of resources in an interactive visual refinement process, and supporting better access to digital resources for teaching and learning about architecture.
- **Society.** Widespread availability of maps of all of science has the potential to dramatically improve the communication of scientific results to the general public.

While different stakeholders might need access to different datasets and might prefer different visual renderings of the data, it is highly desirable to have a well-defined, common science classification system. A visual representation of the classification system via a science base map is beneficial for visual comparison and interpretation. Here we list eight desirable features for a map of science classification system.

1. Use highest quality/coverage data to generate the science map classification system. It is preferable to use paper-level data covering all areas, as using journal level data or highly cited papers exclusively lead to a distorted structure of science [22].
2. Employ advanced dimensionality reduction techniques to map a high dimensional sematic space to a two-dimensional map that preserves the most important data structures [23].
3. Select a clustering and layout that has easy to read, distinct clusters, e.g., subdisciplines, which have about the same number of records, are disjoint (i.e., they do not overlap or occlude one other), and have meaningful labels to ease data interpretation and communication. The map must match the typical viewer’s mental model of the domain.
4. Use graphic design (color, shape, size coding) and legend that can be understood by a large audience. The classification system and map must empower users to form new hypotheses and get new answers.
5. Support interactivity, e.g., zoom, filter, details on demand [24]. Multi-level maps, e.g., two-levels comprising subdisciplines aggregated into disciplines, can support studies at different levels of aggregation.
6. Define a mapping process to classify new data and overlay it onto the map, e.g., journals based on journal names and other records, e.g., patents, funding data based on keywords. As users have a hard time with fractional associations/counting, each record should be associated with one or few subdisciplines.
7. The science map and classification system should be easy to update to capture the continuously evolving structure of science. Computational workflow should be well documented so that is easy to understand in principle and can be replicated by other experts. Updates should preserve the main structure of the map as much as possible.
8. Alignment and comparison of any new science map and classification with commonly used science classifications (e.g., classifications used by Thomson Reuters’ databases, Elsevier’s Scopus, the Library of Congress, Universal Decimal Classification)and the translation of major ontologies into different languages (Science-Metrix, [25].

Subsequently, we describe the methods applied and results achieved when designing and updating the UCSD map of science classification. In section 4, we will use the above features for evaluation.

**Table 1:** Journal coverage of 5-year and 10-year UCSD map of science.

#Journals	5-Year Map	10-Year Map	Difference
**WoS**	9,499	13,520	4,021
**Scopus**	14,789	22,253	7,464
**WoS & Scopus**	15,849	25,258	9,409

## Methods and Results

The design of a map of science and classification system requires data selection, cleaning, analysis, layout, and interpretation. This section details the workflow used to create the original 2005 UCSD map and classification system using Elsevier’s Scopus and Thomson Reuters’ Web of Science (WoS) Science, Social Science, Arts & Humanities Citation Indexes for the years 2001–2005. The original map generation aimed to create a locally and globally accurate basemap of science [26]. Next, we discuss the two independent map updates that were ultimately combined to generate the 2010 UCSD map. The update adds six years (2005–2010) of WoS data and three years (2006–2008) from Scopus to the existing category structure–about 25,000 source titles in total. It aims to increase the coverage of the map, e.g., by adding journals that came into existence after 2005, while preserving the main structure of the map. Finally, we describe the current usage of the updated 2010 UCSD map in research services and commercial tools.

**Figure 4:**
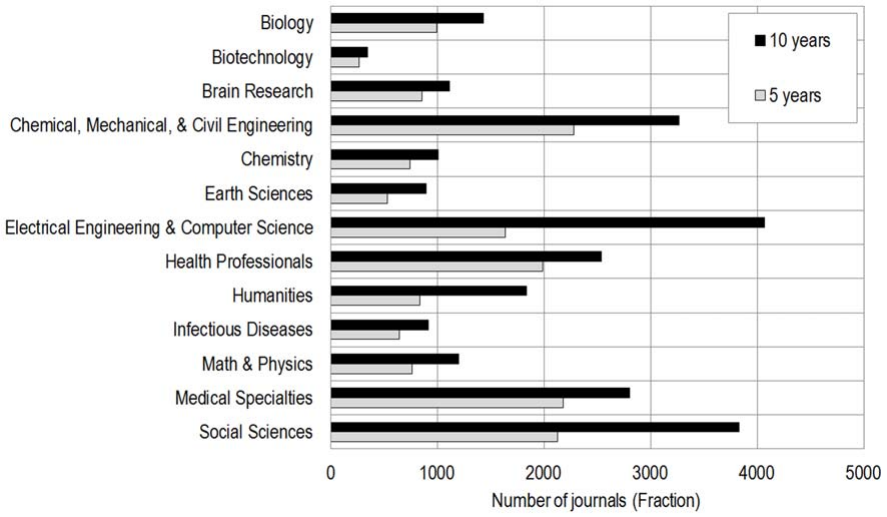
Number of journals per discipline for 5-year (grey) and 10-year (black) UCSD science map.

**Figure 5:**
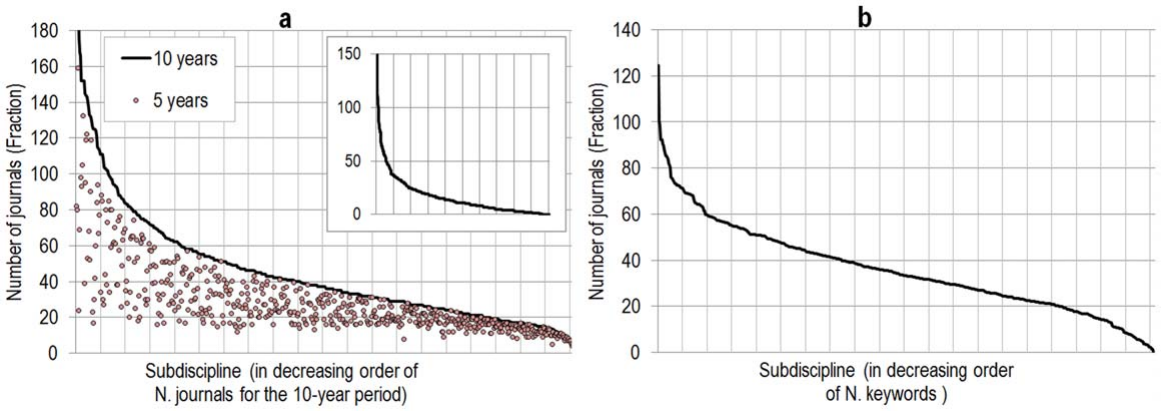
Number of journals per subdiscipline for 5-year (grey/red circles) and 10-year (black line) UCSD science map. Inset: distribution of the gain in number of journals for each subspecialty (a). Number of (fractionally assigned) terms per 554 subdisciplines (b).

### 3.1 Design of the Original 2005 UCSD Map

The UCSD map of science was created by SciTech Strategies in spring 2007 in response to a request by the University of California San Diego (UCSD). UCSD was interested in measuring research leadership in different areas of science. Commonly, existing journal classification systems, such as the Thomson Reuters subject categories, are used to calculate the number of publications and citations per subject area and per person/institution. However, the Scopus database had recently become available, and it had greater coverage (about 15,000 source titles) than the combined Thomson Reuters (Science, Social Science, Arts & Humanities) Citation Indexes (about 9,000 source titles). The decision was made to create the first journal classification system and associated map of science that would comprise the superset of sources from the Thomson Reuters and Scopus sources with improved coverage and greater detail [26]. We intended this classification system and map to become standards and a great deal of care was taken in designing the methodology to create the map. A multistep process, similar to but more involved than the processes used for our previous journal maps [9], [23], was used:

1. Data sources were chosen (Thomson Reuters: 2001–2004 and Scopus: 2001–2005) and source titles (hereafter called journals) from the two data sources were matched and unified.
2. Journal-journal similarity matrices were computed separately for each source (Thomson and Scopus), year and feature type (cited references, keywords) combination.
3. Sums were computed for each matrix, and these sums were used to create weighting factors for each matrix. Eighteen individual matrices from (2) were combined into a single journal-journal matrix.
4. K50 (modified cosine) similarity values were generated for this combined matrix.
5. Multipoint journals (those that are most likely to distribute across multiple categories) were identified and removed from the matrix.
6. The similarity matrix was filtered to reduce the number of non-zero cells to the top-n per journal.
7. This filtered similarity file was used as input to two rounds of graph layout with clustering, resulting in a set of 554 journal clusters. Multipoint journals were added back into the solution, fractionally assigned to the appropriate clusters. Each cluster (subdiscipline) was manually labelled using the journal titles as input. The assignments of journals to these 554 subdisciplines comprised the new UCSD journal classification system.
8. Cluster-cluster similarities were calculated and the clusters were each assigned positions using a combination of graph layout and subsequent scaling and projection of the layout. The UCSD map is the visual depiction of the positions of the 554 clusters (subdisciplines) and the dominant relationships (edges) between them.

The result is a map of 554 journal clusters laid out using a three-dimensional (3D) Fruchterman-Reingold layout in Pajek [27], see Figure 1 (left). A two-dimensional (2D) Mercator projection can be seen in Figure 1 (middle). Subdisciplines were aggregated into 13 high level disciplines based on natural visual groupings within the map and assigned names and colors (see table in Supplement S1).

The process description above is not nearly specific enough to allow replication of the journal classification system or the map. Additional detail for each step above is thus provided in Supplement S1. Although the details are in Supplement S1 rather than here, two of the steps above are noteworthy in that represent significant advances in the state-of-the-art in creating journal classification systems and maps. First, step (3) generates eighteen different matrices which were then reduced to a single matrix. The method of matrix weighting and reduction was designed to use the best information available for each journal pair, and to not penalize journal pairs when information was not available in a particular matrix. It was also designed to weight the influences of cited references (0.8) and keywords (0.2) in a way that is consistent with our experience around the relatively ambiguities of the two feature types. Second, the UCSD map is, to the best of our knowledge, the only science map that has been created from a Riemannian perspective. The use of a sphere as the layout surface presumes no Cartesian boundaries (left, right, top, bottom), and allows a continuous linkage structure to emerge.

Note that many of the decisions discussed above and in Supplement S1 are based on decades of experience gained from working with publication data and running science of science studies. This paper aims to make the current map generation process as transparent as possible. However, more work is needed regarding algorithm selection, parameter and threshold choices, and the visual design of the map to continuously optimize accuracy and legibility of the UCSD map of science and classification system.

### 3.2 Updates Performed to Create 2010 UCSD Map

In the time since its original creation, the 2005 UCSD map has been updated twice, independently, by two different groups. Here we cover the history and details of those two updates.

#### Scopus update

In 2009 the 2005 UCSD map was only being used in two places–at SciTech Strategies, and in Elsevier’s SciVal Spotlight product (see UCSD Map Deployment section). Scopus was indexing many more source titles than it was in 2005. An update was thus needed to bring the coverage of the classification system up to date. SciTech Strategies did this update in June 2009, adding 7,464 new source titles (2006–2008) from Scopus to the existing category structure. This was done by first identifying all new journals that were not in the existing classification system, and then assigning each new journal to one of the existing categories. We counted the numbers of times journals in each category were referenced by the articles in the new journals. Each journal was assigned to the category that it referenced the most, as long as it cited articles within that cluster a minimum of 10 times. Although this update increased the number of Scopus journals in the classification system by 47%, this only accounted for a 13% increase in the number of articles. The added journals had far fewer articles per journal than those that were in the original classification system.

#### Web of science update

In summer 2009, the Cyberinfrastructure for Network Science Center at Indiana University in collaboration with SciTech Strategies added the UCSD map to its Sci2 tool (see UCSD Map Deployment section) to use as the science base map for data overlays. Many researchers and practitioners use the Sci2 tool to analyze Web of Science data yet the UCSD classification system only included WoS titles from 2001–2004. Thus, an update was needed to include 4,021 new WoS journals that had been added since 2004. In 2011, a WoS update was done collaboratively by the Observatoire des Sciences et des Technologies (Canada) and the Cyberinfrastructure for Network Science Center at Indiana University. In addition, this update was done with a view to devising an easily replicable and frequently repeatable updating process that can be used in the future. The updating process is explained in detail here.

Starting with a listing of 15,849 journals covered by the original UCSD map and access to Thomson Reuters’ Web of Science (WoS) Science, Social Science, Arts & Humanities Citation Indexes, we identified all new journals that were not covered in the original list of 15,849. For each of the 4,021 new journals, we counted the number of citations to/from papers published in that journal to/from each subdiscipline of the original map. Here we define a citation to/from a subdiscipline to mean a citation to (or from) a paper published in a journal that is assigned to that subdiscipline in the original map (even if only fractionally, as in the case of multidisciplinary journals). This yielded for each journal an outgoing and incoming citation count for each subdiscipline of the original map. To account for the fact that some subdisciplines publish more papers than others and that, thus, the probability of citing and being cited by these subdisciplines is greater than for smaller ones, we normalized each of these citation counts by the total number of papers published among all journals assigned (even only fractionally) to that subdiscipline. The top subdiscipline citing/cited was then assigned to these new journals. To ease mapping and map reading, each journal was assigned to exactly one subdiscipline, even highly interdisciplinary journals such as *PLoS ONE*. A close look at multidisciplinary journals in the set of 4,021 new journals reveals that *PLOS ONE* and *SCHWEIZERISCHE MEDIZINISCHE WOCHENSCHRIFT* (Swiss Medical Weekly) have the highest combined relative importance across sub-disciplines, as computed by taking the sum of the citations and references for that journal, normalized for the number of papers published in each sub-discipline. This makes sense as *PLOS One* is like *PNAS* and SMW is a general medical weekly. These two journals are followed by journals that have a rather small number of papers (values are not significant statistically) or experienced major editorial changes, e.g., *JOURNAL OF PHYSICAL CHEMISTRY C* on third place is a recent split off of *JOURNAL OF PHYSICAL CHEMISTRY B* but not a multidisciplinary chemistry journal.

Interested to further simplify the 2010 UCSD map, all 40 journals that were assigned to more than one subdiscipline (see step 5 above) in the original 2005 UCSD map were examined. Given that no comprehensive classification scheme at the level of papers exist, each of the papers published in these multidisciplinary journals are categorized in each of the specialties assigned to these journals, irrespective of the actual specialty to which they belong. Although assigning some journals to multiple subdisciplines seems attractive given the breadth of their topic spaces, we feel it is better to assign each journal to only one subdiscipline from a general use standpoint. We thus undertook to reassign these 40 multidisplinary journals to single categories. This was done by counting the number of citations they received from and the references they made to each of the subdisciplines, both in absolute numbers and normalized by the total number of papers published in these citing/cited subdisciplines. Journal self-citations were also removed. We then manually found cut-off points in the distributions of absolute and normalized citations/references to restrict the assignation to a few subdisciplines. Using this method we were able to singly assign 6 of the 40 multidisciplinary journals. *Science*, *Nature*, the *Lancet, British Medical Journal*, and *Journal of the American Medical Association* are among the exceptions that are still multiply assigned. The resulting map covers 22,005 journals from the recent ten years of WoS data.

We assume that the map will be used for many different purposes and at different levels of aggregation–from mapping career trajectories of single scholars to showing strength and weaknesses of entire nations. Each usage might benefit from a slightly different assignment of journals to sub-disciplines, e.g., how interdisciplinary journals are handled. However, the main purpose of a map of science and classification system is its generality and utility for different applications. The update preserves the “visual” structure of the original 2005 UCSD map of science and classification system making it possible to compare data overlays generated with either of the two maps.

#### Combining both updates

In Fall 2011, both updates of the original 2005 UCSD map were combined resulting in the 2010 UCSD map of science classification system that covers ten years of WoS data and eight years of Scopus data and a total of 25,258 journals.

Each journal is assigned to one or more of the 554 subdisciplines. Each subdiscipline is assigned to exactly one of the 13 disciplines; it has a set of keywords; and it has an *x* coordinate (latitude, horizontally) and a *y* coordinate (longitude, vertically) so that it can be mapped spatially. Details on data format and naming schema can be found in Supplement S2.

New data can be science-located, i.e., assigned to a subset of the 554 subdisciplines, via journal names or keywords. Journal name look-up tables are provided for Scopus and WoS data, i.e., nearly all data downloaded from these two sources can be mapped. The number of records mapped is typically depicted by the size of the subdiscipline nodes that corresponds to the average number of papers per year over all years the journal existed between 2001 and 2010. Figures 2 and 3 show exemplary data overlays.

Note that in the UCSD map of science and classification system, no (sub)discipline is more important than another. Like any other classification system, e.g., the Library of Congress Classification or the ACM Classification Hierarchy, there are classes that have more instances. While it seems desirable to arrive at a science map and classification system where each subdiscipline has about the same size (notice that there are multiple alternatives on how to define size, e.g., number of fractionally assigned journals/papers/patents/scholars) this conflicts with the interest to arrive at a map of science and classification system that is locally and globally accurate.

### 3.3 UCSD Map Deployment

Although the UCSD map of science was originally laid out as a spherical map, it is the 2D Mercator map and circular science map that are in most common use. The Mercator map has been incorporated into the Sci2 tool [28] and the VIVO International Researcher Networking software [29], while the circular science map is used as the visual basis for displaying university and country competency maps in Elsevier’s SciVal Spotlight™. Here we discuss both deployments.

The *one-dimensional (1D) circular map* (Figure 1, right) further reduces the complexity of the UCSD map (Figure 1, middle) while preserving the key structure of the 2D map. It corresponds to a view from the “south pole” of the 3D map (Figure 1, left). In addition, the circular map aligns with the consensus map (15 major categories aligned in a structure which is circular with loops) derived from analysis of the structure of 20 different maps of science [8]. It orders the 13 disciplines according to the one-dimensional Riemannian consensus map from that work. Subdisciplines within each discipline, and journals within each subdiscipline are ordered separately using factor analysis. For example, for Chemistry (blue), it was expected that the majority of the associations between journal clusters would be found within a set of five continuous regions. Thus, the journal clusters from Chemistry and from the two bounding regions on each side (pink, purple, cyan, brown), along with their cluster-cluster similarities, were used as input to a factor analysis. Journal clusters in the Chemistry region were ordered based on their loadings along the dominant factor. This same process was used for all thirteen colored regions of the map, resulting in an ordering of all 554 journal clusters around the circle. An example of how the circular map can be used to show institutional competencies is shown in Figure 2. Details about how those analyses are done can be found in Kosecki, Shoemaker & Baer [30] and Klavans & Boyack [26].

The *2010 UCSD map of science and classification system* has been integrated into the Science of Science (Sci2) Tool [28] and the VIVO International Researcher Networking software [29]. The Sci2 tool is a modular toolset specifically designed for the study of science. It supports the temporal, geospatial, topical, and network analysis and visualization of scholarly datasets at the micro (individual), meso (local), and macro (global) levels. It can be freely downloaded from http://sci2.cns.iu.edu, is well documented at http://sci2.wiki.cns.iu.edu. It is widely used in scientometrics research, education and practice with adoption by major US funding agencies such as the National Science Foundation, the National Institutes of Health, the US Department of Agriculture, and the National Oceanic and Atmospheric Administration. Figure 3 (top) shows a sample science map visualization of publication data. The VIVO International Researcher Network uses semantic web technology to facilitate the discovery of researchers and collaborators across the country. Institutions can download VIVO from http://vivoweb.org/download, fill it with high quality institutional data, and participate in the network. VIVO also provides simple temporal, topical, and network visualizations that answer what, when, and with whom questions respectively [31]. Specifically, questions such as: “How many papers were published by an organization or individual over the last years”; “Given a specific scholar, who are his/her main collaborators?” or “What expertise profiles do specific individuals or organizations have and how do they compare to each other?” can be answered. Science maps are employed to help answer the last question. VIVO users can browse the organizational hierarchy of an institution, e.g., Indiana University, and request individual expertise profiles at any level, see Figure 3 (bottom). Alternatively, they can compare up to three organizations, e.g., the School of Informatics and Computing and the School of Library and Information Science.

Most applications of any science map are limited to use of a single database for practical purposes. The UCSD classification system and map give the user an option to use either or both of these two comprehensive bibliographic sources. Both updates are included in the data files distributed with this paper.

The 2010 UCSD map of science and classification system is distributed under the Creative Commons, Attribution-NonCommercial-ShareAlike 3.0 Unported (CC BY-NC-SA 3.0) license (http://creativecommons.org/licenses/by-nc-sa/3.0/). Data format, data dictionary, and usage conditions are detailed in Supplement S2.

### 3.4 Key Features and Comparison

Additional work has been done to compare the WoS update with the original UCSD map of science to show how the updated map meets the ‘Desirable features of a science map’ listed in section 2. We choose to further characterize the WoS update since it will likely have much wider usage than the Scopus update due to inclusion of the map in the Sci2 and VIVO tools.

Table 1 lists the number of journals from WoS and Scopus that are covered by the 5-year and 10-year UCSD map of science. Exactly 4,021 new WoS journals were added to the original map. Also shown are 7,008 journals from the Scopus-based update, see Section 3 and Figure 1 (right). Note that although the addition of new titles is important from the standpoint of completeness and essential for science-locating new journals, its effect on the map layout is relatively small from a numbers perspective. Of the 1,885,278 Scopus records from 2008 (using Scopus XML data from late 2009), only 219,711 (11.65%) are associated with the added titles. Thus, over 88% of the content associated with the journal classification system and map are from the original rigorous mapping process.

As one could expect, the addition of new journals changes the relative importance disciplines and subspecialties in the map. Figure 4 presents the number of journals per 13 disciplines for the 5- and 10-year maps. It shows that the coverage of social sciences (social sciences had a 80% increase, humanities a 119% increase, medical (32%) and natural science (74%)) journals. As a consequence, the relative importance of social sciences and humanities has increased from 19% of journals in the 5-year map to 35% in the 10-year map. Similarly, Figure 5 (left) shows–unsurprisingly–that journals are not distributed evenly in the various subspecialties, and that some subspecialties are more importantly affected by this increase–although most subspecialties only experience a small increase (inset), which suggest that the 5-year map underestimated the research output of these subspecialties. It is also worth noting that the addition of these journals, as well as the realignment of the journals that were in more than one subspecialty, resulted in a more even distribution of journals per subspecialty, as the coefficient of variation–a normalized measure of the dispersion of the distributions–is lower for the 10-year distribution of journals than for the 5-year distribution of journals. There are a total of 66,759 unique terms associated with the 554 subdisciplines. The number of terms per subdiscipline ranges from 16–184. Almost all terms are fractionally assigned and the combined score of terms per subdiscipline ranges from 0.69–124.5, see Figure 5 (right).

#### Comparison with desirable properties

In Section 2 we presented a listing of eight desirable features for generating maps of science. We use these criteria here to review the 10-year UCSD map of science.

1. The new map uses the most recent data from the two most comprehensive bibliographic data providers in existence today: WoS and Scopus. There exist many opportunities for adding other data, e.g., Google Scholar or Google books, conference publications from Citeseer, etc. However, the current two datasets do cover a majority of research published in journals. Plus, the data is of high quality, particularly if compared with datasets that are crawled from the web.
2. As has been shown in previous publications, the UCSD map of science uses dimensionality reduction techniques that preserve the most important structure [23].
3. The map has to be learned–just like the geographical map of our world. It is a two level map–13 color-coded and labelled disciplines and 554 subdisciplines with distinct x,y positions–that supports the generation and exploration data overlays at two levels.
4. A simple layout of subdiscipline nodes connected by links is used for the base map. Data overlays utilize simple color, shape, and size coding. The Sci2 tool provides a legend while the VIVO online map has tool tip information.
5. The map has been deployed online to support interactivity, e.g., zoom, filter, details on demand, see VIVO example. VIVO also facilitates studies at different levels of aggregation, here discipline level and subdiscipline level.
6. New data can be ‘science located,’ i.e., overlaid on the map, based on journal names or keywords associated with a record. All new journals were assigned to exactly one subdiscipline to minimize the confusion caused by fractional associations/counting. Only few highly disciplinary journals–including *Nature, Science, and PNAS*–are associated with multiple subdisciplines.
7. This paper documents the [23] workflow applied to create the 5-year map and to update it at a level of detail that enables experts to replicate map generation. The map update preserves the main structure of the map, including the number of disciplines and subdisciplines. Successive maps can be animated over time to communicate the continuously evolving structure of science. Currently, the number of subdisciplines is fixed but it might be altered in future updates.
8. To ease mapping of new data, lookup tables of cleaned journal names provided with the 10-year UCSD map of science and journal names as a user would download them from WoS and Scopus are provided. An alignment of map structure with commonly used science classifications, e.g., classifications used by Thomson Reuters Databases, Elsevier’s Scopus, the Library of Congress, or ScienceMetrix (http://www.science-metrix.com/OntologyExplorer), is desirable, but has not been done.

## Discussion

Current work aims to study the usability of the UCSD map of science and classification system using formal and informal usability studies conducted within the VIVO project and the Sci2 tool development effort. Different user groups–ranging from novice to expert–are expected to read and interpret the map in different ways and to use it for different purposes. Another line of research aims to ensure the accuracy of maps of all sciences at local and global levels. In [32] a wide range of citation based similarity measures were examined. All of them are symmetrical, which is problematic as a citation from a *Science* or *Nature* paper probably counts more than one from a lowly ranked journal. Boyack, Börner, and Klavans [23] and Boyack, Newman, Duhon, Klavans, Patek et al. [33] examined the need for citation data when generating accurate maps of science. However, using only citation links as a measure for similarity is imperfect as citations follow social networks, depend on the age of a publication, and the number of citations previously received. A combination of linkage and topical data seems desirable. The 2010 UCSD map of science is based on both linkage and keyword data, and thus meets this desire. Recent work by Boyack and Klavans refocuses from the analysis of clusters of journals to analyses of clusters of articles [22], [26]. We anticipate further updates of the 2010 UCSD map of science classification system and plan to develop a more robust versioning system.

## Supporting Information

Supplement S1 (DOC)

Supplement S2 (DOC)
