# Supplementary material for: Design and Update of a Classification System: The UCSD Map of Science
Source: PLoS One. 2012 Jul 12;7(7):e39464. doi: 10.1371/journal.pone.0039464 (PMC3395643; doi:10.1371/journal.pone.0039464)
Supplement: Supplement S1 — (DOC) [file pone.0039464.s001.doc]

Design and Update of a Classification System:
The UCSD Map of Science

Running head: The UCSD Map of Science

Katy Börner^1^, Richard Klavans^2^, Michael Patek^2^, Angela M. Zoss^1^, Joseph R. Biberstine^1^, Robert P. Light^1^, Vincent Larivière^1,3,4^, and Kevin W. Boyack^5^

^1^ Cyberinfrastructure for Network Science Center, School of Library and Information Science, Indiana University, 10th Street & Jordan Avenue, Wells Library, Bloomington, IN 47405, USA

^2^ SciTech Strategies, Inc., Berwyn, PA, 19312, USA

^3^ École de bibliothéconomie et des sciences de l’information, Université de Montréal, C.P. 6128, Succ. Centre-ville, Montréal QC, H3C 3J7, Canada.

^4^ Observatoire des Sciences et des Technologies (OST), Centre Interuniversitaire de Recherche sur la Science et la Technologie (CIRST), Université du Québec à Montréal, C.P. 8888, Succ. Centre-Ville, Montréal, QC H3C 3P8, Canada

^5^ SciTech Strategies, Inc., Albuquerque, NM 87122, USA

**Supplement S1**

***Creating the 2005 UCSD Map of Science Classification System: Details on each of the eight process steps***

1. We desired to create a map that was based on more than one data source and more than one year of data. Article level data from ISI (2001-2004) and Scopus (2001-2005) were available to us, and were used for this exercise. We did not have access to the 2005 ISI data, but feel that this was not a serious issue given the large scale of the data that were used. Journal information from the two sources was matched electronically using journal titles and/or ISSN codes where possible. By-hand matching was done to add additional obvious matches. Only those journals that had at least 20 papers with two or more references or keywords in any particular year were considered. The combined data was comprised of 15,849 unique journals, 9,499 of which were in ISI and 14,789 of which were in Scopus. Of these, 8,439 were common to both data sources.
2. As we have created science maps over the years we have focused on creating the most accurate maps possible . A great deal of work went into deciding how to calculate the similarity measure for this UCSD map. Normalizations such as cosines are commonly applied to journal-journal count matrices. But we were concerned that the orders of magnitude differences between counts for different journals might lead to size effects that were not sufficiently mitigated by simple cosines. Thus, we investigated a variety of frequency modifications that could be used to discount (or augment) individual bibliographic coupling counts, and thus help to mitigate size effects. Using the methods in Klavans & Boyack we compared five different discounting effects: 1, 1/P, 1/P^2^, 1/log(P+1), 1/Plog(P+1), where P=n(n-1)/2 and n is the number of times a particular reference was cited. We found that 1/log(P+1) gave the best results in terms of accuracy vs. coverage over a set of 3,200 journals that are found in both Thomson and PubMed. A combined bibliographic coupling over references and MeSH terms gave very similar results with little degradation in accuracy. We thus chose to generate journal-journal similarities using bibliographic coupling of both references and keywords. (Bibliographic coupling on keywords is done by using keywords as if they were references.) Eighteen separate matrices were calculated, one for each combination of data source, year, and feature type (e.g., ISI 2001 references). We applied the appropriate 1/log(P+1) discount to each count and summed these discounted frequencies by journal-journal pair to create modified frequency matrices.
3. The eighteen matrices were then combined through a series of steps to produce a single matrix using the following protocol.
   1. The set of journals occurring in both ISI and Scopus databases was defined as a control set. Modified frequency sums based on this control set were calculated for each matrix and used to generate weighting factors (see Table 2). Although we used both references and keywords in the calculations they were not weighted equally; an 80:20 ratio between sum-cites and sum-kw was specified to give greater weight to the references. Weights were set such that [sum-cites*wt-cites + sum-kw*wt-kw] would be the same for each data source plus year combination (e.g. ISI 2001 would have the same overall control set weight as Scopus 2001, Scopus 2002, etc.)
   2. Each matrix in (2) was modified by its weighting factor.
   3. Cites and keyword matrices were combined for each data source, year combination by simply adding cell values, resulting in nine matrices.
   4. Within each year the ISI and Scopus matrices were combined by selecting the “best” value on a cell-by-cell basis. These “best” values were specified as
      1. If both matrices have values, use the maximum of the two values. The basis for this is the assumption that if the values differ, the combined matrix should be based on the data source with the highest information content relative to the journal pair.
      2. If only one matrix contains a value, use that value.
      3. If neither matrix contains a value for that year, the cell is NULL.
      4. One exception was used: in the 2005 calculation, if there was no Scopus value for a cell and the ISI 2004 matrix contained a value, the ISI value was used.
   5. The five annual matrices resulting from the above calculation were averaged. Only cells containing non-zero or non-null values were included in the average. Zeros were not included in the averages and were thus not penalized.
4. The k50 (modified cosine) normalization was applied to the combined matrix. K50 is simply the cosine index minus the expected value of the cosine index for each cell in the matrix. Positive values indicate a larger than expected similarity; negative values indicate the opposite.
5. Multipoint journals are those that are very hard to classify numerically into a single category. They couple strongly to journals from multiple categories, cannot be unambiguously assigned based on citation patterns, and thus have the potential to overaggregate a journal cluster solution. We chose to identify and remove these journals from the calculation so that they would not overly impact the structure of the solution. The number of positive k50s values by journal were counted, journals were sorted by descending count, and these values were plotted by rank order. The resulting scree plot was examined and a clear break occurred at 40 journals. These 40 journals (see Table 3) were specified as multipoint journals and their values were removed from the k50 matrix.
6. The k50 matrix was further filtered, keeping only the top- *n* highest similarity values for each journal. We varied the *n* in top- *n* from 5 to 15, scaling its value to log(sum-cites) by journal. One might think that this reduction in information would result in a loss of accuracy. However, previous studies have shown that filtering the similarities can actually increase the accuracy of the cluster solution . From an anecdotal point of view, given that authors only consider submitting a particular article to a handful of journals (in other words, they only consider dominant relationships), there seems to be no reason to include the long tail of journal similarities in a journal mapping calculation.
7. Journals were clustered using the filtered k50 matrix. DrL (now OpenOrd), a graph layout routine that uses a random walk process and prunes edges based on degree and edge distance, was run using the default cutting parameter of 0.8. Long edges between nodes of high degree are preferentially pruned. A DrL run at default cut will typically prune around half the input edges. Using the output journal positions (x,y) and uncut edges, an average-linkage clustering algorithm then assigns journals to clusters . Two successive levels of clustering[[1]](#footnote-2) were used to generate the final solution of 554 journal clusters. To keep clusters from becoming too large, only those level 1 journal clusters with fewer than 100 journals were entered in the second round of clustering. The 40 multipoint journals were then added back into the solution, and were fractionally assigned to those clusters receiving at least 1% of their citations.
8. The 554 journal clusters were assigned positions (so that they could be viewed in a visual map) using a multistep process. First, cluster-cluster similarities were calculated by summing modified frequencies (see step 2) to the cluster level, and then calculating k50. This cluster-cluster k50 matrix was then further filtered to top-*n* similarities using the approach in step 6. This filtered similarity file was then subjected to a 3D Fruchterman-Reingold layout in Pajek. Upon examination, the resulting 3D layout was found to have much in common with a spherical solution – none of the 554 clusters was near the centroid. A transform was then imposed on this solution that, using the centroid as a basis, placed every cluster on a unit sphere (radius=1) while maintaining their radial coordinates (see Figure 1). A 2D version of the map was then generated as the Mercator projection (see Figure 2) of the 3D map. Names were manually assigned to each journal cluster using the journal titles as input. The clusters were manually grouped into 13 high level categories based on natural visual groupings within the map and cluster names (see Table 4).

**Tables Section**

**Table 2:** Weighting factors

|  | **ISI Cites** | **ISI Kw** | **Scopus Cites** | **Scopus Kw** |
| --- | --- | --- | --- | --- |
| 2001 | 2.261 | 0.604 | 2.229 | 0.388 |
| 2002 | 2.239 | 0.608 | 2.186 | 0.385 |
| 2003 | 1.955 | 0.533 | 2.016 | 0.352 |
| 2004 | 2.062 | 0.541 | 1.879 | 0.349 |
| 2005 |  |  | 1.821 | 0.339 |

**Table 3:** Original multipoint journals

| ANAL_BIOANAL_CHEM | FOREST_ECOL_MANAG | NATURE |
| --- | --- | --- |
| ANAL_CHEM | GEOPHYS_RES_LETT | NEUROIMAGE |
| ANAL_CHIM_ACTA | HYDROBIOLOGIA | PEDIATRICS |
| ANGEW_CHEM_INT_EDIT | IEEE_T_BIO-MED_ENG | PHYS_REV_E |
| ANN_NY_ACAD_SCI | IND_ENG_CHEM_RES | P_NATL_ACAD_SCI_USA |
| APPL_ENVIRON_MICROB | JAMA-J_AM_MED_ASSOC | P_SOC_PHOTO-OPT_INS |
| ATMOS_ENVIRON | J_ACOUST_SOC_AM | RISK_ANAL |
| BRIT_MED_J | J_AGR_FOOD_CHEM | SCIENCE |
| CHEMOSPHERE | J_AM_CHEM_SOC | SCI_TOTAL_ENVIRON |
| ENVIRON_HEALTH_PERSP | J_BIOL_CHEM | SOC_SCI_MED |
| ENVIRON_MANAGE | J_CHROMATOGR_A | WATER_RES |
| ENVIRON_MONIT_ASSESS | J_THEOR_BIOL | WATER_SCI_TECHNOL |
| ENVIRON_SCI_TECHNOL | LANCET |  |
| ENVIRON_TOXICOL_CHEM | LECT_NOTES_COMPUT_SC |  |

**Table 4:** High-level categories and colors

| **Category** | **Color (Pajek)** |
| --- | --- |
| Math & Physics | Purple (Mulberry) |
| Chemistry | Blue (Blue) |
| Engineering | Cyan (SkyBlue) |
| Earth Sciences | Brown (Mahogany) |
| Biology | Green (OliveGreen) |
| Biotechnology | Teal (Emerald) |
| Infectious Diseases | Dark Red (BrickRed) |
| Medical Specialties | Red (Red) |
| Health Services | Orange (Peach) |
| Brain Research | Light Orange (Dandelion) |
| Humanities | White (White) |
| Social Sciences | Yellow (Yellow) |
| Computer Science & Electrical Engineering | Pink (Lavender) |

1. Average-link threshold values of 3.563 and 2.843 were used for the 1^st^ and 2^nd^ level cluster solutions, respectively. [↑](#footnote-ref-2)
