# Supplementary material for: Design and Update of a Classification System: The UCSD Map of Science
Source: PLoS One. 2012 Jul 12;7(7):e39464. doi: 10.1371/journal.pone.0039464 (PMC3395643; doi:10.1371/journal.pone.0039464)
Supplement: Supplement S2 — (DOC) [file pone.0039464.s002.doc]

Design and Update of a Classification System:
The UCSD Map of Science

Running head: The UCSD Map of Science

Katy Börner^1^, Richard Klavans^2^, Michael Patek^2^, Angela M. Zoss^1^, Joseph R. Biberstine^1^, Robert P. Light^1^, Vincent Larivière^1,3,4^, and Kevin W. Boyack^5^

^1^ Cyberinfrastructure for Network Science Center, School of Library and Information Science, Indiana University, 10th Street & Jordan Avenue, Wells Library, Bloomington, IN 47405, USA

^2^ SciTech Strategies, Inc., Berwyn, PA, 19312, USA

^3^ École de bibliothéconomie et des sciences de l’information, Université de Montréal, C.P. 6128, Succ. Centre-ville, Montréal QC, H3C 3J7, Canada.

^4^ Observatoire des Sciences et des Technologies (OST), Centre Interuniversitaire de Recherche sur la Science et la Technologie (CIRST), Université du Québec à Montréal, C.P. 8888, Succ. Centre-Ville, Montréal, QC H3C 3P8, Canada

^5^ SciTech Strategies, Inc., Albuquerque, NM 87122, USA

**Supplement S2**

***Distributing the 2010 UCSD Map of Science: Data Format, Data Dictionary, and Usage Conditions***

The 2010 UCSD map of science and classification system covers ten years (2001-2010) of data from Thomson Reuters’ Web of Science and eight years (2001-2008) of Elsevier’s Scopus, specifically the fractional assignment of about 25,000 journal names to 554 subdisciplines grouped into 13 disciplines of science. The counts for major record types are given here:

1. 13 disciplines with labels and color codes
2. 554 subdisciplines with x, y positions and size
3. 15,849 journals captured by 5-year map
4. 25,258 journals captured by 10-year map
5. 13,520 journal names used by Thomson Reuters
6. 22,253 journal names used by Scopus
7. 21,630 Scopus journal ID numbers
8. 19,988 ISSN numbers
9. 66,759 terms

Note that items number 1.-4. are used for rendering the base map of science; while data listed under 5.-10. are needed to science locate and overlay data based on journal name, ISSN number lockup, Scopus ID, or common term usage. Note that the journal names used by Thomson Reuters and Scopus are not unique, i.e., multiple journal names might be mapped onto one journal in the 5-year map or the 10-year map.

The map is freely available online at <http://sci.cns.iu.edu/ucsdmap>. This supplement details the data formats in which the map is provided as well as conditions under which this map can be freely used for research, education, and commercial applications.

***Data Format***

The 2010 UCSD map of science classification system is shared as MS Excel spreadsheets and MS Access database. Both formats conform to the table schema depicted in Figure 6 and the data dictionary given in Table 5.

The table schema was designed to maintain information on journals, their relations to the sub-disciplines and how those relations change over time. It has nine tables and the content and utility of these tables is explained subsequently.

Crucial to the creation of a base map is the assignment of *journal_names* to *subdisciplines* that are further assigned to *disciplines*. The two *arch** tables are used to keep track of past and current versions of journal to *subdisciplines* mappings. The most current, mapping from journals to subdisciplines is kept in the *subd_journal* table for convenience, while the description of older mappings, such as the 2005 UCSD map are stored in the archive table. This keeps the data available for tracking how relations have changed over time while minimizing the chance of the old relations being used in error.

Vital to the usage of the base map is an effective means to ‘science locate’ new data, e.g., via journal names or terms used in the text of new records. One of the greatest challenges when using journal names is the various ways that a journal’s name can be written. With that in mind, tables for names and ISSNs (called *journal_names* and *journal_issn*) were established using data from major publishers, here Scopus and WoS. This allows for a variety of abbreviations and alternate names to be mapped to the same journal, as well as allowing for information to be output using the nomenclature of the researcher’s choice (ISI, Scopus, etc.). When analyzing non-journal publication data, e.g., patents or news records, journal names cannot be used to science locate data. Here, terms extracted from titles or abstracts can be matched against the *subd_terms* table to identify relevant subdiscipline(s).

In order to visualize the base map of science or to generate data overlays, a network of (sub)discipline nodes and edges needs to be created using multiple tables. Node names and coordinates can be found in the *subdisciplines* table, while node positions, colors, and discipline names are drawn from the *disciplines* table. Subdiscipline node size can be taken from the *subdiscipline* table or be calculated from the data, e.g., number of papers or citations a scholar received in a certain (sub)discipline. Base map node sizes and edges are given in a .net file. Edges can be added based on citations between papers from different (sub)disciplines, co-author relations, related terms or whatever other metric the researcher deems appropriate.

The table schema of the MS Access database is given in Figure 6. The data can also be accessed as five xls MS Excel spreadsheets. The networked structure of the 554 subdisciplines is distributed via two .net data files. One file has information on the 554 subdisciplines and their linkages exclusively while the other .net file has 13 additional nodes—one for each discipline.

*1) Subdiscipline to discipline look-up table*

This six column table names and associates the 554 subdiscipline IDs used in ‘*Journal to subdiscipline table’* to proper discipline names. X and y reflect the location of the node within the science map. Subdiscipline size in 6^th^ column is computed as the sum of all fractional assignments and is used for node size in ‘*Network file for map layout’*.

subd_id | subd_name | disc_id | x | y | size

*2) Discipline look-up table*

This four column table names and links the 13 discipline IDs used in the *‘Subdiscipline to discipline look-up table’*. X and y reflect the location of the discipline node within the science map. Color is the color used for all nodes for that discipline.

disc_id | disc_name | x | y | color

*3) Edge list table*

This four column table defines the list of edges in the map of science. Each line describes one edge, with the two subdisciplines it links, the weight of the edge and the color used to draw it. If an edge connects two subdisciplines within the same discipline, it is drawn in the color of that discipline. If the subdisciplines connected are from different disciplines, the edge is drawn in gray.

subd_id1 | subd_id2 | weight | color

*4) Journal to subdiscipline look-up table*

This four column table lists the names of the more than 22,000 journals and assigns their unique journal IDs to the 554 subdiscipline IDs. Some journals are fractionally assigned to multiple subdisciplines. The sum of jfraction over each journal equals 1.

journ_id | formal_name| subd_id | jfraction

*5 - 7) Journal name conversion table to science locate new publication data via journal names*

Journal names in data retrieved from publishers, the web or personal bibliographies will contain errors. These three files aim to capture common errors but will by no means be able to capture all possible misspellings. The first file contains the unique journal ID as used in ‘*Journal to sub-discipline table’* along with all known ISSNs associated with that journal as well as the Scopus journal ID, if available. The second file contains the unique journal ID and any Scopus IDs associated with that journal. The third file contains the unique journal ID and all names by which that journal has been listed. Common source types like ISI and Scopus are marked as appropriate.

journ_id | issnjourn_id | scopus_id

journ_id | journal_name | source_type

*8) Journal to subdiscipline table (2005 UCSD map)*

This table provides the journal-subdiscipline mapping for the 2005 UCSD map of science originally published by Boyack and Klavans.  Like the*’Discipline look-up table’*, this four column table lists the names of the almost 16,000 journals and assigns their unique journal IDs to the 554 subdiscipline IDs. Some journals are fractionally assigned to multiple subdisciplines.  The sum of jfraction over each journal equals 1 in most instances, however 49 journals were erroneously assigned to two subdisciplines at full value.

journ_id | formal_name| subd_id | jfraction

9) *Keyword look-up table to science locate non-publication data*

This table associates terms to the 554 subdiscipline IDs used in ‘*Journal to subdiscipline table’*. Some terms are fractionally assigned to multiple subdisciplines. The tfraction ranges from 0 to 1 for a subdiscipline/term pair.

subd_id |term | tfraction

*10) Network file for map layout*

This file is provided in the Pajek .net data file format, see format details in (http://pajek.imfm.si). Each node represents one of the 554 subdisciplines, has an ID (also used to decide order of rendering), the subdiscipline number as labels in quotes, x and y position, size (same for x_fact and y_fact). Node color corresponds to color of associated discipline (see ‘*Subdiscipline to discipline look-up table’*).

Order of rendering, “subdiscipline#”, x, y, size (same for x_fact and y_fact), node color, ring color

553 " 122" 6.12 -0.38 x_fact 11.3 y_fact 11.3 ic Canary

554 " 381" 6.12 -0.38 x_fact 11.7 y_fact 11.7 ic Canary*edges

1 2 5.20 c Yellow

1 3 5.20 c Yellow

1 5 5.20 c Yellow

Edges are color coded as follows: If two nodes are the same color, the edge between them is also coded that color. Edges between nodes of different colors are gray.

When size coding nodes according to data overlays it is advisable to place larger nodes first and then smaller nodes to minimize occlusion.

Node sizes were computed using paper counts as provided by Scopus and ISI, with Scopus given priority due to its more selective nature. These paper counts were mapped in a linear fashion to a 1-20 scale that was used as the value for the disc size.

A second .net file has been available that includes nodes for the 13 disciplines. These nodes are located at the center of their respective subdisciplines, with a very small actual node (0.1) and an appropriate label.

***Data Dictionary***

The naming scheme is designed for modularity. Any name repeated in the schema is a foreign key from the master table to the child table. The root tables are disciplines, journals and *archived_maps*. All columns that end in “*_id*” are unique numeric identifiers created as part of the database. All columns that end in “*_name*” are the text name of the described element. The columns that end in “*fraction*” are for the mapping of one element to another (such as journals to subdisciplines). For each of these columns, the sum over the element being mapped should be equal to 1, that is, the entirety of the element should be mapped, but only once. All other fields are properties of the element being described.

***Usage Conditions***

The 2005 and the 2010 UCSD map of science classification systems are shared under the Creative Commons, Attribution-NonCommercial-ShareAlike 3.0 Unported (CC BY-NC-SA 3.0) license (<http://creativecommons.org/licenses/by-nc-sa/3.0/>). That is, you are free to share, e.g., to copy, distribute and transmit the work, and to remix, i.e., to adapt the work under the following conditions:

- Attribution — You must attribute the work in the following manner (but not in any way that suggests that they endorse you or your use of the work): Cite this paper and use the following acknowledgment text: *“The authors wish to acknowledge The Regents of the University of California, SciTech Strategies, Observatoire des Sciences et des Technologies, and the Cyberinfrastructure for Network Science Center for making the 2010 UCSD Map of Science and Classification System available for this work."*
- Noncommercial — You may not use this work for commercial purposes.

If you alter, transform, or build upon this work, you may distribute the resulting work only under the

same or similar license to this one.

**Figure 6:** UCSD map table schema, see large version at <http://sci.cns.iu.edu/ucsdmap/data/UCSDmapDBSchema.pdf>

**Table 5:** UCSD map data dictionary.

| **Table/Column Name** | **Data Type** | **Key?** | **Description** | **Comment** |
| --- | --- | --- | --- | --- |
| **Disciplines** |  |  | The master table for information about the 13 disciplines |  |
| disc_id | integer | Primary | A unique numeric identifier assigned to each discipline |  |
| disc_name | varchar(100) |  | The name of the discipline | unique |
| X | double |  | x-coordinate of the discipline node on the map of science |  |
| Y | double |  | y-coordinate of the discipline node on the map of science |  |
| Color | varchar(25) |  | The color usually used to display nodes and edges related to the discipline in maps of science | unique |
|  |  |  |  |  |
| **Subdisciplines** |  |  | The master table for information about the 554 subdisciplines |  |
| subd_id | integer | Primary | A unique numeric identifier assigned to each subdiscipline |  |
| subd_name | varchar(100) |  | The name of the subdiscipline | unique |
| disc_id | integer | Foreign | The discipline that the subdiscipline is a part of | linked to disciplines.disc_id |
| x | double |  | x-coordinate of the subdiscipline node on the map of science |  |
| y | double |  | y-coordinate of the subdiscipline node on the map of science |  |
| size | double |  | size of the subdiscipline node on the map of science |  |
|  |  |  |  |  |
| **edges** |  |  | The master table of edges drawn in the base map of science |  |
| subd_id1 | integer | Primary | The first subdiscipline node that the edge links | linked to subdisciplines.subd_id |
| subd_id2 | integer | Primary | The second subdiscipline node that the edge links | linked to subdisciplines.subd_id |
| weight | double |  | The weight of the edge |  |
| color | varchar(100) |  | The color of the edge |  |
|  |  |  |  |  |
| **journals** |  |  | The master table for information about journals |  |
| journ_id | integer | Primary | A unique numeric identifier assigned to each journal |  |
| formal_name | varchar(250) |  | The unique name of the journal that is considered the formal name |  |
| yr_started | integer |  | The year the journal started publication |  |
| publisher | varchar(100) |  | The publisher of the journal |  |
| scopus_id | varchar(100) |  | The Scopus Journal ID for the publication. If more than one is used, they will be separated by a “/”. |  |
|  |  |  |  |  |
| **subd_journal** |  |  | The linking table defining the current mapping between journals and subdisciplines |  |
| journ_id | integer | Primary | A unique numeric identifier assigned to each journal | linked to journals.journ_id |
| subd_id | integer | Primary | A unique numeric identifier assigned to each subdiscipline | linked to subdisciplines.subd_id |
| jfraction | double |  | The fraction of the journal that is linked to the subdiscipline | the sum of these should add to one for each journal |
|  |  |  |  |  |
| **arch_subd_journal** |  |  | The linking table defining older or alternative mappings between journals and subdisciplines |  |
| journ_id | integer | Primary | A unique numeric identifier assigned to each journal | linked to journals.journ_id |
| subd_id | integer | Primary | A unique numeric identifier assigned to each subdiscipline | linked to subdisciplines.subd_id |
| jfraction | double |  | The fraction of the journal that is linked to the subdiscipline | the sum of these should add to one for each journal |
| map_id | integer | Primary | A unique numeric identifier assigned to each journal/subdiscipline mapping | linked to arch_maps.map_id |
|  |  |  |  |  |
| **arch_maps** |  |  | The master table for information about older or alternative journal/subdiscipline mapping |  |
| map_id | integer | Primary | A unique numeric identifier assigned to each journal/subdiscipline mapping |  |
| map_desc | varchar(250) |  | A brief description of the mapping, including the source and timeframe |  |
|  |  |  |  |  |
| **subd_terms** |  |  | The table associating author-generated terms with subdisciplines |  |
| subd_id | integer | Primary | A unique numeric identifier assigned to each subdiscipline | linked to subdisciplines.subd_id |
| term | varchar(100) | Primary | An author-generated term |  |
| tfraction | double |  | A score assigned to the term/subdiscipline association |  |
|  |  |  |  |  |
| **journal_names** |  |  | A table of known names associated with a given journal |  |
| journ_id | integer | Primary | A unique numeric identifier assigned to each journal | linked to journals.journ_id |
| journal_names | varchar(250) | Primary | A known name variant for this journal |  |
| source_type | varchar(100) |  | The type of name and where it might be found regularly (WoS, Scopus, citations, common typo) |  |
|  |  |  |  |  |
| **journal_issn** |  |  | A table of ISSNs associated with a given journal |  |
| journ_id | integer | Primary | A unique numeric identifier assigned to each journal | linked to journals.journ_id |
| issn | varchar(25) | Primary | An ISSN that identifies this journal |  |
| issn_details | varchar(100) |  | The details about this ISSN, when it was used, what version of the journal it applies to, etc |  |

**Figure Legends**

**Figure 6:** UCSD map table schema, see large version at <http://sci.cns.iu.edu/ucsdmap/data/UCSDmapDBSchema.pdf>

**Tables Section**

**Table 5:** UCSD map data dictionary
